# Amiodarone-induced thyrotoxicosis in a child with congenital heart disease

**DOI:** 10.1530/EDM-26-0048

**Published:** 2026-08-24

**Authors:** Pooja Karandikar, Rebecca Carter, Sudhir Vashist, Paula G Newton

**Affiliations:** University of Maryland Department of Pediatrics, Baltimore, Maryland, USA

**Keywords:** amiodarone, thyroid, thyrotoxicosis, amiodarone-induced, iatrogenic

## Abstract

**Summary:**

Amiodarone-induced thyrotoxicosis (AIT) is a known complication of amiodarone usage in adults that occurs secondary to unregulated overproduction of thyroid hormone due to excess iodine exposure in an abnormal thyroid gland (type one), the release of stored thyroid hormone due to a direct cytotoxic effect on a normal thyroid gland (type two), or both (mixed type). Therapy for AIT depends on the subtype present and typically consists of discontinuation of amiodarone and the addition of thyrostatic drugs or corticosteroids. AIT is a very uncommon adverse side effect of amiodarone use in children. We discuss a pediatric patient with a history of complex congenital heart defects and ventricular arrhythmia treated with amiodarone who developed AIT. This case illustrates the importance of regular thyroid function monitoring with clear guidelines, especially for pediatric patients, and the need for improved education regarding this phenomenon.

**Learning points:**

- Pediatric patients requiring amiodarone are at high risk of both amiodarone-induced hypothyroidism (AIH) and amiodarone-induced thyrotoxicosis (AIT).
- Although AIT is rare in pediatrics, it requires special attention due to the unique challenges in diagnosis and management.
- Pediatric patients managed with amiodarone require close monitoring of the thyroid function levels at a minimum of every six months and for up to two years after amiodarone is discontinued, and more frequently if symptoms of hypothyroidism, hyperthyroidism, or other complications develop.
- Treatment of AIT with steroids per the standard of care can increase the risk of iatrogenic adrenal insufficiency, and patients should be monitored for signs of adrenal insufficiency.

## Background

Amiodarone is a class III antiarrhythmic medication that blocks voltage-gated potassium channels in cardiomyocytes and is used to treat supraventricular and ventricular tachyarrhythmias (1). It is a structural analog of thyroid hormone; therefore, thyroid dysfunction is one of the most common adverse effects of amiodarone therapy, and its lipophilicity allows it to concentrate in adipose tissue, the thyroid gland, and cardiac and skeletal muscle (2). The effects of this dysfunction can persist for months after discontinuation due to its lipophilic structure, 37% iodine composition, and its half-life of 40–100 days (3, 4). In pediatric patients, this amiodarone-induced thyroid dysfunction has unknown ramifications on growth and neurocognitive development and has the potential to worsen cardiac arrhythmia, which underscores the importance of consistent monitoring of thyroid function when taking this medication (3).

Two types of amiodarone-induced thyroid dysfunction patterns may occur: amiodarone-induced hypothyroidism (AIH) and amiodarone-induced thyrotoxicosis (AIT). AIT is approximately half as common, and cases are even less frequent among pediatric patients. The development of AIH may be the result of the high iodine content in amiodarone, causing decreased production of T4 and T3 by downregulating thyroid peroxidase and the sodium-iodine symporter (3). This process mimics the protective ability of the healthy thyroid gland to react to exposure to excess iodine, known as the Wolff–Chaikoff effect. Direct inhibition of iodothyronine 5′-deiodinase may also play a role.

There are two possible mechanisms for AIT. In AIT type one, patients typically have a known history of thyroid disease or autonomous thyroid function, such as Grave’s disease, and the excess iodine from amiodarone can lead to overproduction of thyroid hormone. This is also known as the Jod-Basedow phenomenon (4). These patients can present with symptoms of hyperthyroidism or thyroid enlargement earlier than those with AIT type two and are treated with thyrostatic drugs, such as methimazole, thiamazole, or propylthiouracil. Alternatively, AIT type two develops due to the dose-dependent cytotoxic effects of amiodarone, resulting in the release of stored thyroid hormone. This type can be self-limiting but can also be treated with oral steroids (3). A mixed type of AIT also exists in cases where the disease shows traits of both type one and type two. In these cases, patients are treated simultaneously with steroids and thyrostatic medications. Type one and type two can be distinguished by using a variety of methods, including color flow Doppler sonography of the thyroid, radioactive iodine uptake scans, the presence of thyroid disease history, the level of thyroid antibodies, and the response to treatment (5).

We describe a 13-year-old child with a complex past medical history, including ventricular arrhythmias and a successfully resuscitated sudden cardiac arrest treated with an automatic implantable cardioverter defibrillator (AICD) and amiodarone, presenting in florid thyrotoxicosis based on routine thyroid studies prior to a revision of the AICD. Their case was complicated by the need for a prolonged course of oral steroids.

## Case presentation and investigation

The patient is a 13-year-old child with a history of dextrocardia, pulmonary atresia, and levo-transposition of the great arteries with ventricular inversion who had surgical repair at six months of age complicated by coronary insufficiency, ventricular dysfunction, and myocardial ischemia. At seven years of age, there was a sudden cardiac arrest that was successfully resuscitated, and they subsequently underwent an epicardial biventricular AICD implant. Given ventricular arrhythmia and baseline prolonged corrected QT interval, they were started on beta blockers. At 11 years of age, they were readmitted with worsening ventricular arrhythmia and AICD system lead malfunction despite beta blocker therapy; therefore, amiodarone was started. At 13 years of age, AICD lead malfunction recurred. They were hospitalized for revision of their AICD system.

During their admission, thyroid studies revealed thyrotoxicosis, with a thyroid stimulating hormone (TSH) level of <0.01 mIU/L (reference range (RR): 0.5–4.5 mIU/L), free T4 of 5 ng/dL (RR: 0.6–2.5 ng/dL), and total T3 level of 175 ng/dL (RR: 97–169 ng/dL). Additional thyroid function and antibody studies showed a thyroid stimulating immunoglobulin (TSI) of <0.10 IU/L (RR: ≤0.54 IU/L), TSH receptor-binding antibody of <0.80 IU/L (RR: ≤1.75 IU/L), thyroid peroxidase antibody of 0.4 IU/mL (RR: 0.0–9.0 IU/mL), and thyroglobulin antibody of <0.9 IU/mL (RR: 0.0–4.0 IU/mL). The patient denied tachycardia, heat intolerance, abdominal pain, nausea, vomiting, difficulty swallowing, sleep disturbance, weight loss, change in appetite, or hair loss. They complained of diarrhea for a few days 2 months prior to admission, but that had resolved. On examination, the heart rate was 62, the blood pressure was 92/54, the patient was afebrile, and there was no goiter, palpable thyroid nodules, proptosis, or exophthalmos. Previous thyroid studies performed nearly ten months prior had been within normal limits. The amiodarone was discontinued, and they transitioned to sotalol due to their thyrotoxicosis. AICD revision was postponed as thyrotoxicosis could complicate their surgery and worsen their ventricular arrhythmia. Given the high risk of inappropriate shock from lead malfunction, AICD therapies were deactivated and LifeVest therapy was instituted.

## Treatment

To manage the thyrotoxicosis, they started on a course of 40 mg oral prednisone daily for suspected AIT type two. Pre-treatment cortisol levels were normal at 8.3 μg/dL (RR: 4.46–22.7 μg/dL). After five days, there was mild improvement in their total T3 level to 141 ng/dL, and they were discharged on LifeVest therapy with plans of AICD revision surgery once their thyrotoxicosis was better controlled. The prednisone was gradually weaned to 15 mg, although they did complain of more noticeable fatigue, dizziness, nausea, and heat intolerance.

At the endocrinology follow-up ten days after discharge, the patient presented with thyroid enlargement, worsening heat intolerance, insomnia, headaches, and tremor. They had been evaluated in the ED for orthostatic hypotension and near-syncope 6 days prior to the visit and released. They did not have thyroid nodules, tenderness, or asymmetry. A trial of methimazole 10 mg was initiated due to concern for a mixed type of AIT, but it was discontinued a few weeks later due to mild transaminitis and worsening thyrotoxicosis with the steroid wean. The prednisone dose was increased to 20 mg daily. A thyroid ultrasound was obtained due to concern for mixed type AIT and was notable for a right lobe measuring 4.7 × 1.4 × 1.6 cm, a left lobe measuring 4.5 × 1.2 × 1.8 cm, and the isthmus measuring 0.2 cm. The thyroid gland was of normal size and echogenicity. There were no masses, nodules, or hypervascularity. The thyrotoxicosis persisted for several more weeks without definitive improvement, and due to persistent symptoms of hyperthyroidism, the prednisone was increased to 30 mg daily to stabilize them for AICD revision surgery.

More than nine weeks after their hospitalization, the prednisone dose was further increased to 40 mg due to continued signs of AIT, with TSH < 0.005 mIU/L (RR: 0.45–4.5 mIU/L) and free T4 level 3.77 ng/dL (RR: 0.93–1.60 ng/dL), and unrelenting heat intolerance, insomnia, tremor, fatigue, dizziness, and hypotension that worsened with inadequate fluid intake.

## Outcome and follow-up

**Figure 1:**
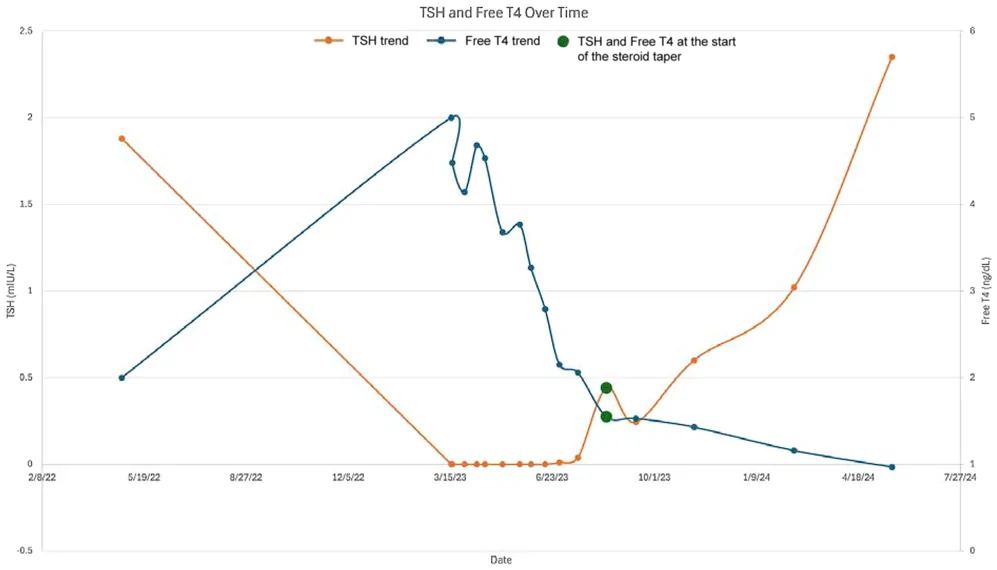
TSH and free T4 measurements were recorded over the prednisone treatment period from March 2023 to April 2024. The prednisone taper was initiated in August 2023. Baseline results from April 2022 are included as well as results from one month after completing the taper in May 2024. TSH reference range: 0.5–4.5 mIU/L. Free T4 reference range: 0.6–2.5 ng/dL.

Four months after hospitalization, with better control of their thyrotoxicosis, their epicardial AICD revision was completed without complications. Once they recovered from surgery, they began a slow prednisone taper tailored according to their symptoms and tolerance over six to nine months. They continued to experience intermittent dizziness, lightheadedness, hypotension, insomnia, and fatigue. After nearly nine months, their thyroid hormone levels stabilized, with a TSH of 0.601 mIU/L (RR: 0.45–4.5 mIU/L), free T4 of 1.43 ng/dL (RR: 0.93–1.6 ng/dL), and total T3 of 140 ng/dL (RR: 71–180 ng/dL). A year later, their cortisol level was 9.9 μg/dL (RR: 6.2–19.4 μg/dL) and a cosyntropin stimulation test subsequently showed a normal cortisol response, indicating recovery of their adrenal function (Fig. 1).

## Discussion

Despite its side effect profile, amiodarone is a very effective antiarrhythmic agent for the treatment of both supraventricular and ventricular tachyarrhythmias (4). With thyroid dysfunction reported in nearly 15–20% of patients, AIH is twice as common as AIT, and available pediatric data suggest that AIT is far less common in this population with a prevalence of approximately 2%. Despite its low prevalence in the pediatric population, AIT is a serious and potentially life-threatening side effect, especially in vulnerable patients with underlying cardiac abnormalities leading to potential worsening of cardiac tachyarrhythmia and increased mortality. Thus, early diagnosis and effective management are critical to ensure optimal outcomes. Additionally, amiodarone may mask some of the clinical manifestations of thyrotoxicosis, such as tachycardia, further making the diagnosis difficult. When considering the pediatric population, there is limited guidance and education on the use of thyroid function monitoring after initiating treatment with amiodarone (3). The timeframe in which symptoms of thyroid dysfunction typically develop can be widely variable among pediatric patients, with some studies proposing that symptoms develop between three and ten months after starting treatment, while others suggest that dysfunction can present up to two years after discontinuing treatment (6, 7).

Our patient developed thyrotoxicosis after management with amiodarone for more than two years, which is a longer latency period than reported in other case reports involving pediatric patients. It took nearly nine months for their thyroid hormone levels to normalize after discontinuation of the drug and initiation of steroid therapy. Some studies support thyroid monitoring every three to six months and up to one year after discontinuation of amiodarone, while others suggest that longer monitoring is required since some pediatric patients develop asymptomatic thyroid dysfunction (6, 7). The age of amiodarone initiation may have also affected the subtype of thyroid dysfunction they developed, emphasizing the unpredictable range in symptoms and thus in the type of treatment required (5). Interestingly, our patient required nearly 13 months of steroid therapy, a prolonged course compared to other cases, which increased the risk of developing secondary adrenal insufficiency (2, 5, 8). When managing amiodarone-induced thyroid dysfunction with steroids, it is crucial to slowly taper steroids to reduce the risk of adrenal crisis and to monitor for symptoms of ongoing adrenal insufficiency.

This case sheds light on the variability in which AIT presents, as well as the complexity in its management. Although there is a need for further study of this complication in the pediatric population, routine thyroid function testing while taking amiodarone and for at least two years after its discontinuation may be beneficial to capture the broad array of presentations and prevent the development and long-term ramifications of undiagnosed amiodarone-induced thyroid dysfunction.

## Declaration of interest

The authors declare that there is no conflict of interest that could be perceived as prejudicing the impartiality of the research reported.

## Funding

This research did not receive any specific grant from any funding agency in the public, commercial, or not-for-profit sector.

## Patient consent

Written informed consent for publication of their clinical details and/or clinical images was obtained from the guardian of the patient.

## Author contribution statement

PK is the primary author. RC, SV, and PGN all contributed to the study design and writing and editing of the manuscript. SV and PGN were responsible for the diagnosis and management of the patient. PGN is the senior and corresponding author. All authors reviewed and approved the final draft.
